# Two Behavioral Tests Allow a Better Correlation Between Cognitive Function and Expression of Synaptic Proteins

**DOI:** 10.3389/fnagi.2018.00091

**Published:** 2018-04-04

**Authors:** Marta Balietti, Giorgia Fattorini, Arianna Pugliese, Daniele Marcotulli, Luca Bragina, Fiorenzo Conti

**Affiliations:** ^1^Center for Neurobiology of Aging, INRCA, IRCCS, Ancona, Italy; ^2^Department of Experimental and Clinical Medicine, Section of Neuroscience and Cell Biology, Università Politecnica delle Marche, Ancona, Italy; ^3^Fondazione di Medicina Molecolare, Università Politecnica delle Marche, Ancona, Italy

**Keywords:** brain aging, cognitive decline, hippocampus, synaptic proteins, synaptotagmin 1, Morris water maze test, step-through passive avoidance test

## Abstract

The molecular substrate of age-associated cognitive decline (AACD) is still elusive. Evidence indicates that AACD is related to synaptic impairment in hippocampus, but different hippocampal regions play different roles, with the dorsal hippocampus (DH) associated to spatial learning, and the ventral hippocampus (VH) crucial for emotionality. If changes in hippocampal function contributes to AACD, this contribution may be reflected in alterations of synaptic protein levels. A commonly used approach to investigate this issue is western blotting. When this technique is applied to the entire hippocampus and the cognitive impairment is evaluated by a single task, changes in expression of a protein might undergo a “dilution effect”, as they may occur only in a given hippocampal region. We show that two behavioral tests yield more accurate results than one test in evaluating the function of the whole rat hippocampus by studying the expression of synaptotagmin 1 (SYT1), a vesicular protein whose expression in aged hippocampus is reportedly inconsistent. Analysis of SYT1 levels in the whole hippocampus of rats selected by the Morris water maze (MWM) test only failed to highlight a difference, whereas analysis of SYT1 levels in the whole hippocampus of rats categorized by both the MWM and the step-through passive avoidance (STPA) tests demonstrated a significant increase of SYT1 level in impaired rats. These findings, besides showing that SYT1 increases in impaired aged rats, suggest that using the whole hippocampus in blotting studies may prevent false negative results only if animals are categorized with tests exploring both DH and VH.

Memory dysfunction is a hallmark of brain aging. Although numerous studies have been focused on this phenomenon over the last decades (Park et al., 2002; Salthouse, 2003, 2012; Butler et al., 2004; Dixon and De Frias, 2014; Pudas et al., 2014), the molecular substrates of age-related cognitive deficits are still elusive.

Mounting evidence indicates that age-associated cognitive decline (AACD) can be related to synaptic impairment in hippocampus (e.g., Vanguilder and Freeman, 2011; Vanguilder et al., 2011), and investigations on synaptic structure and function and on synaptic proteins have flourished in the recent past (e.g., Burke and Barnes, 2006; Bishop et al., 2010; Bano et al., 2011). Indeed, the hippocampus is involved in important aspects of learning and memory (e.g., Bettio et al., 2017), but different hippocampal regions play different roles. In their 1995 article, Moser et al. (1995) showed that removal of 40% of the dorsal hippocampus (DH) impaired spatial learning, whereas removal of 70% of the ventral part did not affect performance in this test. On the contrary, the ventral hippocampus (VH), as part of a network comprising prefrontal cortex, amygdala and subcortical structures associated with hypothalamic-pituitary-adrenal axis, is crucial for emotionality, i.e., fear, anxiety and depression (Kjelstrup et al., 2002; Fanselow and Dong, 2010; McLaughlin and Gobbi, 2012; Bannerman et al., 2014).

If changes in hippocampal synaptic function contribute to AACD, this contribution may be determined by or reflected in alterations of synaptic protein levels. A commonly used approach to investigate such an issue is western blotting. Indeed, it is informative, easy, inexpensive and non-time consuming. However, when this tecnique is applied to the entire hippocampus in order to obtain enough material to study a broad pannel of structurally and functionally linked proteins and the cognitive impairment is evaluated by a single task, significant changes in expression of a given protein might undergo a “dilution effect”, as they may occur only in a given hippocampal subregion underpinnig a specific functional domain (Shimohama et al., 1998; Chen et al., 2007; Cao et al., 2013).

## The Two Behavioral Tests Approach

To overcome this problem, we reasoned that the hippocampal regions analyzed by western blotting, hence the amount of tissue examined and the ability of the method to detect alterations in proteins levels, can be increased by employing a larger number of behavioral tests. Here, we report some methodological considerations gathered in the course of our ongoing investigations on the correlation between the level of synaptic proteins, studied by western blotting, and behavioral analysis. We administrated in series the Morris water maze (MWM) and the step-through passive avoidance (STPA) tests. We considered these tests suitable to validate/reject our hypothesis for three reasons: (i) together, they allow to assess the performance of both the DH and the VH (Moser et al., 1995; Lorenzini et al., 1996; Ambrogi Lorenzini et al., 1997; Wang et al., 2017); (ii) the MWM test is the preferred test for assessing core aspects of spatial learning and memory (Vorhees and Williams, 2014), while one-trial inhibitory avoidance is the best studied task concerning the molecular post-training processes of hippocampal cellular memory consolidation (Izquierdo et al., 2016); and (iii) evidence exists that spatial and aversive memories, both crucial for a proper interaction with the environment, undergo age-related changes (Lovatel et al., 2013; Beaudet et al., 2014; Leffa et al., 2014).

**Figure 1:**
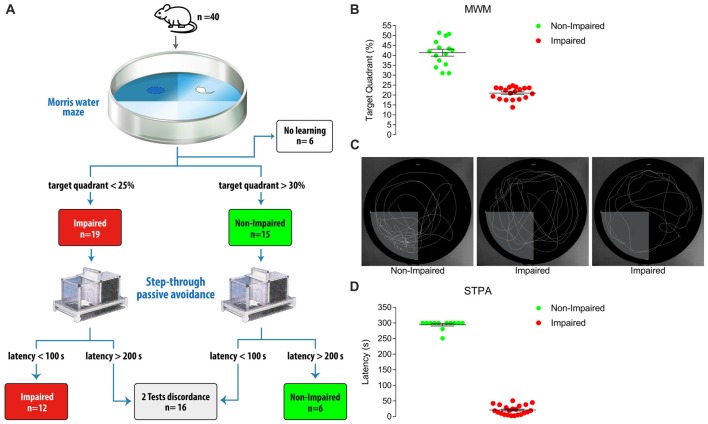
**(A)** Diagram showing animal categorization and numbers. The initial cohort included 40 male Sprague-Dawley albino rats aged 24 months. Performing the Morris water maze (MWM) test according to Vorhees and Williams (2006) yielded 19 Impaired and 15 Non-impaired rats, while 6 rats were excluded as they were unable to significantly reduce the distance moved to find the platform during the 5-day learning phase. These 34 rats were then subjected to the step-through passive avoidance (STPA) test according to Platano et al. (2008). This step produced 12 Impaired and 6 Non-impaired rats according to both tests; 16 rats had opposite result in the two tests, and were excluded. **(B)** Percent time spent in the target quadrant at probe day of the 34 rats classified by the MWM test. Animals were classified as Non-Impaired if the time spent in the target quadrant was >30% and superior to that spent in each non-target quadrant; rats where categorized as Impaired if their permanence in the target quadrant was <25%. To exclude any categorization bias due to visual deficits, a *post hoc* control was applied and the distance moved by Non-Impaired and Impaired rats to find a multiple-located visible platform was compared. **(C)** Examples of performances at probe day. The first panel represents a Non-Impaired rat with a percentage of time spent in the target quadrant of 51; the second and the third panels represent the performance of two Impaired rats with percentages of time spent in the target quadrants of 24 (random swimming equally distributed in all the quadrants) and 17 (preferential searching in non-target quadrants), respectively. **(D)** Latency at test day of the STPA task of the 34 rats classified by the MWM test. A value <100 s to enter the dark compartment 24-h after the shock release was taken to indicate Impairment and a latency >200 s was taken to indicate Non-impairment. Horizontal lines indicate mean ± SEM values.

We first administered the MWM test to 40 old male Sprague-Dawley albino rats (24 months of age). Thirty-four animals were categorized as Impaired (*n* = 19) and Non-impaired (*n* = 15), while six (15%) were excluded because they were unable to learn the task (Figures 1A–C). The addition of the STPA test determined the exclusion of further 16 animals, because we selected only animals that exhibited the same results in both tests. Indeed, only 18 of the 34 rats that were categorized by the MWM test could be included after categorization with the STPA test (12 Impaired, 6 Non-impaired), whereas a further 16 did not comply the inclusion criteria. Altogether, 55% of the cohort was excluded (Figures 1A,D). Therefore, in our experimental conditions, performing two tests implied that less than half of the animals passed the selection and were categorized as Impaired and Non-impaired. Notably, the calculation does not take into account physiological mortality.

**Figure 2:**
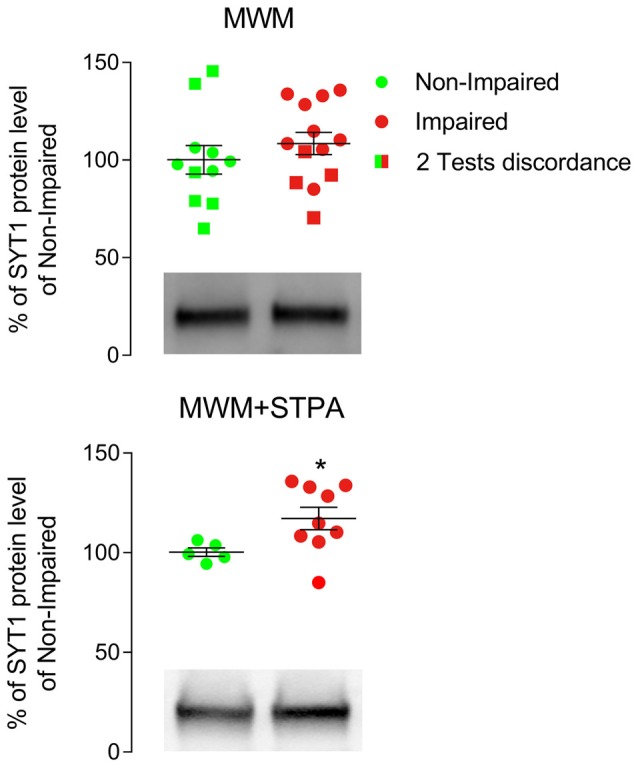
Western blotting analysis of hippocampal tissue from male Sprague-Dawley albino rats categorized only with the Morris water maze (MWM) test or with the MWM and the step-through passive avoidance (STPA) tests (MWM+STPA). Rats were anesthetized with intraperitoneal chloral hydrate (300 mg/kg), decapitated, and the hippocampi were quickly collected. Homogenization and crude synaptic plasma membrane preparation were as in Danbolt et al. (1990) and Marcotulli et al. (2017). The Bio-Rad Protein Assay (Bio-Rad Laboratories GmbH, Munich, Germany) was used to determine the total amount of protein in each homogenate (3–4 measurements/ homogenate). For quantitative analysis, standard curves with increasing total protein concentrations were drawn to define a linear range for immunoblot densitometric analysis (Bragina et al., 2006). For optimal resolution of synaptotagmin 1 (SYT1; 105011 clone 41.1; 1:500; Synaptic System, Göttingen, Germany; RRID AB_887832) concentrations, western blotting analysis was performed in crude synaptic membranes using 7 μg of total protein. Immunoblot densitometric analysis of MWM group rats was conducted in tissue from 11 Non-impaired (green) and 13 Impaired subjects (red; 108.5% ± 5.72% of Non-impaired rats), whereas the MWM+STPA group included 5 Non-impaired (green) and 9 Impaired (red) rats (117.2% ± 5.64% of Non-impaired rats; *p = 0.029, Mann Whitney test). Circles represent animals that were Non-Impaired (green) or Impaired (red) at both MWM and STPA tests, while squares represent animals that had discordant performances at the two tests (i.e., Non-Impaired at MWM test but Impaired at STPA test (green) and Impaired at MWM test but Non-Impaired at STPA test (red)). Horizontal lines indicate mean ± SEM values. Graphs include representative western blottings of Non-Impaired and Impaired animals classified by MWM (top) or MWM+STPA (bottom) tests.

Next, we tested the hypothesis that two behavioral tasks provide more accurate results than one test alone in evaluating the function of the whole rat hippocampus by studying the expression of the vesicular protein synaptotagmin 1 (SYT1). We specifically selected SYT1 because its expression in the aged hippocampus provided inconsistent results to date. Indeed, Chen et al. (2007), who categorized their animals with the MWM test and used only dorsal hippocampal tissue for western blotting analysis, reported that cognitive impairment correlated with increased SYT1 levels, whereas Nicolle et al. (1999), who categorized the animals by the same test but examined the whole hippocampus found no difference in SYT1 levels. In our study, analysis of SYT1 levels in the whole hippocampus of rats selected by the MWM test alone, as in the study of Nicolle et al. (1999), failed to highlight a difference (Figure 2), whereas analysis of SYT1 levels in the whole hippocampus of rats categorized with both the MWM and the STPA tests demonstrated a significant increase of SYT1 level in rats with cognitive impairment (117.2% ± 5.64% vs. Non-Impaired rats; *p* = 0.029, Mann Whitney test; Figure 2). Therefore, using the entire hippocampus to analyze proteins levels requires classifying animals by at least two tests, exploring the dorsal and ventral portion of the hippocampus, if false negative results are to be avoided.

## How to Choose?

Our findings raise the question whether, when investigating cognitive decline, information should be obtained from a single hippocampal sub-region or from the whole structure. The answer to this question is important for the proper interpretation of results. Rats categorization with two behavioral tests allows using the entire hippocampus for western blotting studies, i.e., about 300 μg of protein in crude synaptic plasma membrane preparations (Danbolt et al., 1990; see also Marcotulli et al., 2017). Since 4–5 measurements and 7 μg of protein per measurement are generally required for each antigen, the whole hippocampus allows testing 8–10 antigens. In contrast, the use of the MWM test alone involves that although a larger number of categorized animals is available (meaning that one could employ half the animals that are needed for two tests protocol), only the upper third of the hippocampus should be evaluated (Chen et al., 2007; Cao et al., 2013). This amount of tissue is sufficient to test only 2–3 antigens. If the western blotting study will involve only few proteins, it would be preferable to select animals using exclusively one test, and to analyze only the involved part of the hippocampus, thus allowing to employ fewer animals. If, however, more proteins are to be investigated, the two-tests approach will allow to use all the hippocampal tissue, thus maximizing the ratio between the amount of tissue and the number of animals used.

## Conclusion

Our findings show a relationship between AACD, assessed by the MWM and the STPA tests, and increased SYT1 levels in the whole hippocampus of aged rats. They also suggest that the use of the whole hippocampus in western blotting analysis may avoid false negative results if animals have been categorized with behavioral tests that explore both the DH and the VH. Moreover, these findings might shed new light in the field of cognitive dysfunctions, in particular Alzheimer’s disease. Indeed, synaptotagmins seem to play a role as APP interactors in promoting Aβ generation (Gautam et al., 2015; Kuzuya et al., 2016): the application of the present methodology to assess their level could help to better clarify their possible involvement in Alzheimer’s disease etiopathogenesis.

## Ethics Statement

All experimental procedures involving animals and their care were carried out in accordance with the European Community Council Directive guidelines (2010/63/UE) and approved by the Italian Ministry of Health (code 336/2016-PR).

## Author Contributions

FC and GF conceived the project. MB, GF, AP, DM and LB performed the experiments. MB and GF gathered and analyzed the data. FC supervised the project and discussed the data. GF, MB and FC wrote the article.

## Conflict of Interest Statement

The authors declare that the research was conducted in the absence of any commercial or financial relationships that could be construed as a potential conflict of interest.
